# Standard Protocols for Characterising Primary and In Vitro‐Generated Human Hepatocytes

**DOI:** 10.1111/jcmm.70390

**Published:** 2025-02-05

**Authors:** Zahra Heydari, Roberto Gramignoli, Abbas Piryaei, Ensieh Zahmatkesh, Paria Pooyan, Homeyra Seydi, Andreas Nussler, Dagmara Szkolnicka, Hassan Rashidi, Mustapha Najimi, David C. Hay, Massoud Vosough

**Affiliations:** ^1^ Department of Regenerative Medicine, Cell Science Research Center Royan Institute for Stem Cell Biology and Technology, ACECR Tehran Iran; ^2^ Division of Pathology, Department of Laboratory Medicine Karolinska Institutet Stockholm Sweden; ^3^ Department of Biology and Anatomical Sciences, School of Medicine Shahid Beheshti University of Medical Sciences Tehran Iran; ^4^ Department of Tissue Engineering and Applied Cell Sciences, School of Advanced Technologies in Medicine Shahid Beheshti University of Medical Sciences Tehran Iran; ^5^ Siegfried Weller Institute for Trauma Research University of Tübingen Tübingen Germany; ^6^ Centre for Regenerative Medicine, Institute for Repair and Regeneration University of Edinburgh Edinburgh UK; ^7^ Department of Developmental Biology and Cancer UCL Great Ormond Street Institute of Child Health London UK; ^8^ Laboratory of Pediatric Hepatology and Cell Therapy Institute of Experimental and Clinical Research, UCLouvain Brussels Belgium; ^9^ Experimental Cancer Medicine Institution for Laboratory Medicine, Karolinska Institute Huddinge Huddinge Sweden

**Keywords:** hepatic functional characterisation, hepatocyte‐like cell, in vitro differentiation, in vitro hepatic maturation, pluripotent stem cells

## Abstract

Hepatocyte‐like cells (HLCs) derived from pluripotent stem cells (PSCs) or direct reprogramming are an unlimited source of human hepatocytes for biomedical applications. HLCs are used to model human diseases, develop precise drugs and establish groundbreaking regenerative cell‐based therapies. Primary human hepatocytes are the gold standard for studying human liver biology and pathology. However, their widespread use is limited by their rapid dedifferentiation in vitro, reliance on transplant‐rejected donor organs, poor scalability and significant batch‐to‐batch variations. Therefore, high‐quality ‘off‐the‐shelf’ HLCs are needed to overcome those limitations. Basic stepwise differentiation protocols have been developed to generate HLCs from PSCs. To evaluate the quality of the in vitro generated products, HLCs have been phenotyped using various methods. This review discusses various biological assays and methods available for the robust evaluation of HLC quality, emphasising the importance of using 24‐h cultured primary human hepatocytes (PHHs) as a reference standard for comparison.

## 1 Introduction

Functional hepatocyte‐like cells (HLCs) derived from progenitor or pluripotent stem cells (PSCs) facilitate the study of liver organogenesis and physiology. The use of stem cell–derived HLCs can aid in drug discovery and development of cell‐based therapies for congenital and chronic liver diseases. Over the past two decades, many studies have demonstrated the generation of mature functional HLCs from PSCs or multipotent stem cells. Early protocols for hepatic differentiation and maturation are based on embryoid body formation and spontaneous differentiation using specific growth factors and hormones [1]. To efficiently generate mature functional hepatocytes, undifferentiated stem cells must be exposed to inductive and repressive signals [2]. Such an approach is extremely simple compared to prenatal and postnatal liver organogenesis, requiring only a few weeks of exposure but exhibiting varying differentiation efficiency. Various aspects of mammalian developmental biology are used to improve these processes [3, 4]. In addition to classical two‐dimensional (2D) settings, three‐dimensional (3D) platforms, such as organoid and spheroid generation, have been established to improve the HLC functions and stability. Liver organoids and spheroids derived from adult and PSCs are more scalable than primary human hepatocytes (PHHs) [5, 6, 7, 8], enabling the mass production of functional HLCs for basic and translational research and development [9, 10]. Recent advancements in direct reprogramming techniques have enabled the generation of HLCs from somatic cells, providing an alternative to traditional methods derived from induced pluripotent stem cells (iPSCs). These approaches present unique advantages and challenges; however, both pathways yield cells that require rigorous quality assessment for effective application in research and therapy [11]. However, HLC quality is key to facilitate their application as routine models. Various biological assays are used worldwide to evaluate the quality of in vitro‐generated HLCs. In this review, we discuss the different approaches available for primary hepatocyte phenotypic profiling and propose guidelines to follow for the characterisation of in vitro generated hepatocytes.

### 1.1 Cell Morphology and Ultrastructure Analysis

Primary human hepatocytes preserve their polygonal shape after isolation from the liver and replacement in cell culture. Adherent human hepatocytes contain a granular cytoplasm with several vesicular inclusions and mitochondria. Postnatal hepatocytes are characterised by high levels of polyploidy, which increases with age. Notably, reestablishment of cell polarity, which is essential for proper cell function, is observed in PHH 3D cultures [12].

#### 1.1.1 Light and Fluorescence Microscopy

**FIGURE 1:**
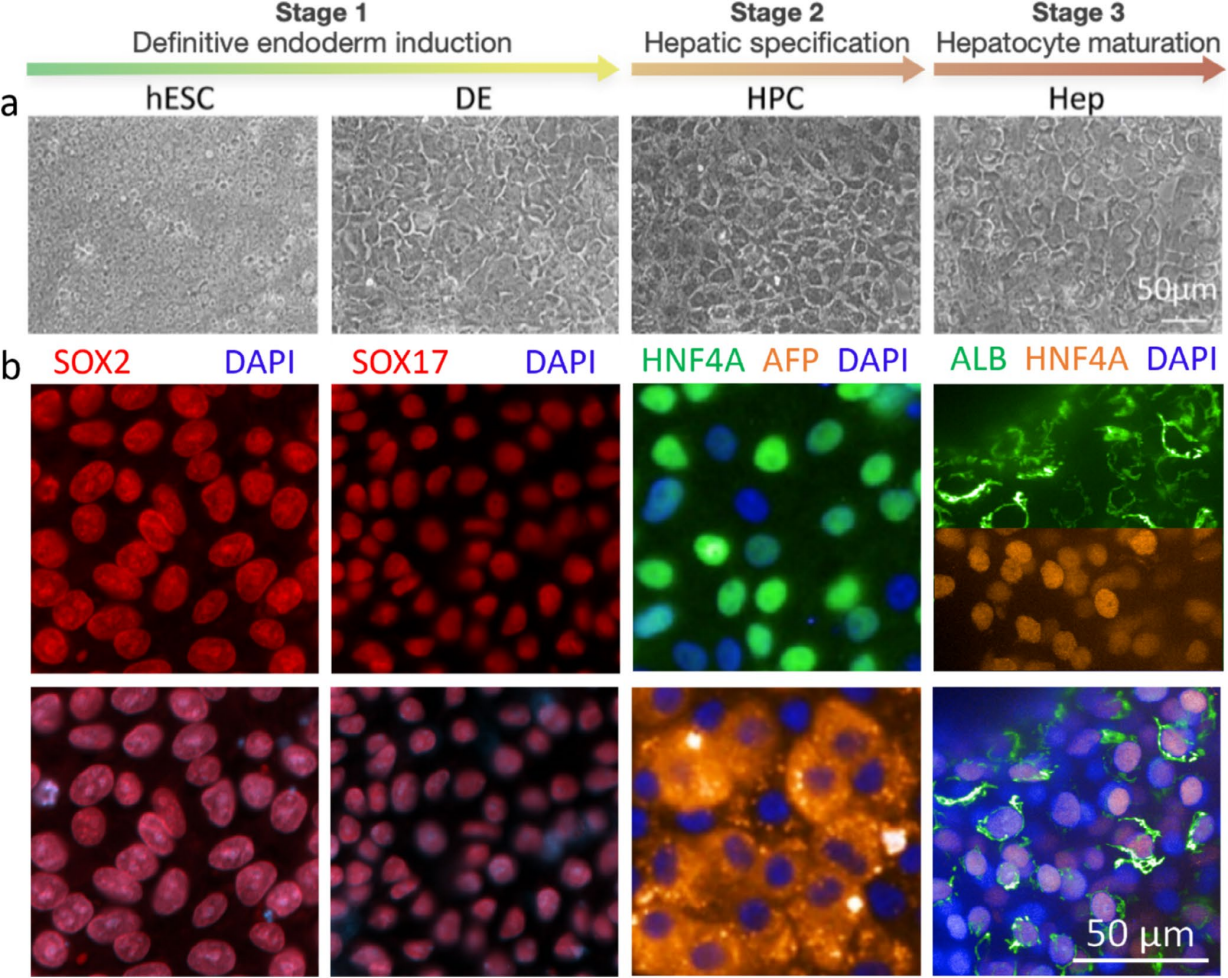
Microscopic imaging of hepatocyte‐like cell (HLC) differentiation. (a) Phase‐contrast images of the cells at key differentiation stages. A polygonal morphology of hepatocytes is observed during the maturation phase. (b) Immunofluorescence staining for pluripotent (SRY‐box transcription factor 2 [SOX2]), definitive endoderm (SOX17) and hepatic (hepatocyte nuclear factor 4 alpha [HNF4α], albumin [ALB] and alpha‐fetoprotein [AFP]) markers counterstained with 4′,6‐diamidino‐2‐phenylindole (DAPI). AFP, alpha‐fetoprotein; ALB, albumin; DE, definitive endoderm; Heps, hepatocytes derived from hESCs; HNF4α, hepatocyte nuclear factor 4 alpha; HPC, hepatic progenitor cell; hPSC, human pluripotent stem cell; SOX, SRY‐box transcription factor.

Characteristic cell morphology can be observed via conventional light microscopy or immunostaining with specific antibodies. Morphological examination using microscopy is the first assessment used to determine the cell health and attachment. Other techniques include phase‐contrast, epifluorescence and laser scanning confocal microscopy [13]. These three imaging platforms provide the resolution required to visualise the cellular and subcellular structures. Despite its advantages, maximum resolution of 0.2 μm is a major limiting factor for light microscopy. Additional limitations of light microscopy are listed in Table 1. Immunofluorescence staining allows researchers to record the dynamics of cells over defined periods (Figure 1). Figure 1 shows the three stages of directed differentiation of hPSCs into HLCs. The morphology of cells at different stages is shown in Figure 1a, and stage‐specific biomarkers are shown in Figure 1b.

**TABLE 1:** Advantages and limitations of light microscopy and transmission electron microscopy (TEM) [13, 14].

Light microscopy	Transmission electron microscopy (TEM)
**Advantages**	
Feasible and simple sample preparation	Higher magnification and resolution
Cell morphology remains intact during preparation	Provides detailed information on the subcellular ultrastructure
Both living and fixed specimens can be studied	Electromagnetic field is used to fine‐tune the magnification
Original colour of the specimens can be observed	High‐quality images
Live imaging is possible	
Targets can be visualised using simple stains	
**Limitations**	
Maximum magnification is approximately 1500×	Live specimens cannot be examined
Disrupted visualisation of 3D structures	Only provides black and white images
Low resolution for thicknesses > 0.2 μm	Artefacts are common due to sample preparation method
	Expensive and time consuming

Epifluorescence microscopy uses different wavelengths of light to target multiple stained molecules, identify cellular components and discriminate between cell subtypes in living and fixed samples [15]. To achieve higher resolution and greater contrast, laser scanning confocal microscopy has been used because it can capture a series of images along the z‐axis (Figure 1b) and allows their 3D reconstruction [15, 16]. Recently, two‐photon microscopy has been used to image bioengineered tissues and large cellular aggregates at a high resolution. However, this type of microscope has several disadvantages, including high cost and phototoxicity in the focal plane. However, this can be reduced through technical adaptations [17].

#### 1.1.2 Transmission Electron Microscopy (TEM)

**FIGURE 2:**
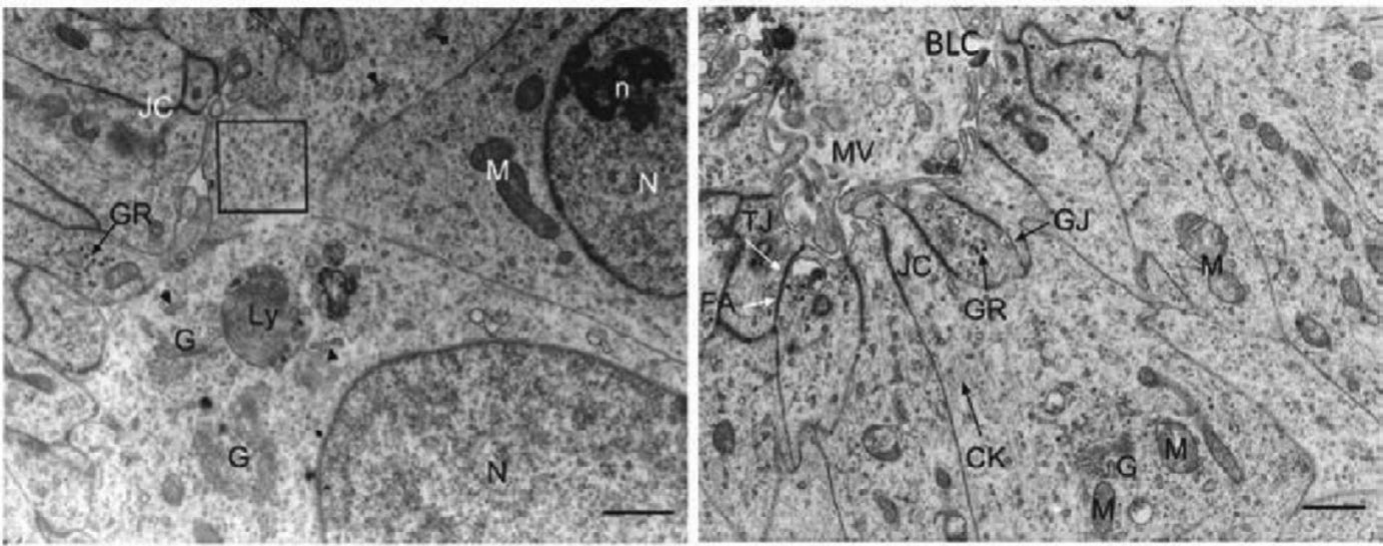
Transmission electron micrographs of day 21 three‐dimensional HLCs differentiated from human pluripotent stem cells (hPSCs). Nucleus (N), nucleoli (n), mitochondria (M), Golgi apparatus (G), lysosomes (Ly), rough endoplasmic reticulum (arrowheads), glycogen granules (GR), intermediate filaments (CK), tight junctions (TJ), gap junctions (GJ), fascia adherens (FA), junctional complex (JC), microvilli (MV) and bile‐like canaliculus (BLC). Scale bar: 1 μm [10]. Adapted from the ‘Generation of functional HLCs from hPSCs in a scalable suspension culture’ by Vosough M, et al. 2013; 22 (20):2693–705; Stem Cells and Development.

Transmission Electron Microscopy is used to detect and characterise cellular and subcellular components at the ultrastructural level with a high resolution of less than 1 nm [18]. In conventional TEM, sections less than 100 nm in thickness were placed in a vacuum column. An accelerated electron beam crossed the sample and was focused by an objective lens to produce an electron micrograph with a maximum magnification of ×10^5^ [19, 20]. These high‐resolution micrographs enable researchers to observe subcellular organelles and analyse cell projections and junctional complexes [21]. Figure 2 presents the ultrastructure of subcellular elements, such as the Golgi apparatus, rough endoplasmic reticulum, glycogen granules, intermediate filaments, tight junctions, gap junctions, microvilli and bile‐like canaliculus. However, TEM has limitations, including its restriction to fixed samples of a defined thickness. Moreover, skilled operators are required to conduct the analyses. The other limitations of the TEM are summarised in Table 1.

#### 1.1.3 Transcriptomic Profiling

Hepatic maturation is governed by selective upregulation and downregulation of different genes in a time‐dependent manner. These genes include stemness‐ and lineage‐specific genes, which are summarised in Table 2 [22, 23]. During definitive endoderm and early hepatic specification/commitment, the genes commonly monitored are SRY‐box transcription factor 17 (*SOX17*), *HEX*, *GATA4*, hepatocyte nuclear factor 4 alpha (*HNF4a*), *HNF3* (*α*, *β* and *γ* forms), *HNF1* and *C/EBP* (*α* and *β*). As hepatic induction continued, early hepatic maturation genes, such as *TTR*, *TAT*, *G6P*, *PEPCK*, *GS* and *ASGPR*, or secretory proteins, such as alpha‐fetoprotein (AFP), albumin (ALB) and alpha‐1 anti‐trypsin (A1AT), were measured. Upon hepatic maturation, the expression of liver‐specific enzymes, such as the cytochrome P450 (CYP) family (*CYP1A1*, *1A2*, *2C8/9/19*, *2D6*, *3A4/7* and *7A1*) and urea cycle mediators (*CPS‐1*, *OTC* and *ASSL*) was detected. In addition, phase 2 enzymes (*UGTs*, *SULTs* and *GSTPs*), transporters (*BSEP*, *MRP2*, *NTCP* and *OATP1*) and clotting factors (*V*, *VII* and *IX*) [22, 24] are also evaluated. Gene expression analysis was performed using RT‐PCR with PHHs or normal liver tissue as a reference [22]. Over the last decade, high‐throughput sequencing platforms have been developed, furthering our understanding of human liver biology. Although informative, qRT‐PCR and RNA‐seq are classically performed in ‘bulk’, and the data represent an average of gene expression patterns through the thousands or millions of cells. This can mask cell‐to‐cell differences within populations. To overcome this obstacle, single‐cell RNA sequencing (scRNA‐seq) has been developed to map gene expression in individual cells at a high resolution [25]. Although transcriptome analysis is a powerful tool, it does not guarantee successful translation into functional proteins. To validate HLCs, gene expression data should be combined with other analyses, including immunostaining and metabolic and proteomic profiling, to improve confidence [26]. By comparing the transcriptomic profiles of HLCs with those of primary human hepatocytes (PHHs) cultured for 24 h, researchers can establish a benchmark for evaluating HLC quality. This comparison is crucial because PHHs cultured for 24 h represent an optimal state of hepatic function before significant dedifferentiation occurs. High‐quality HLCs should exhibit gene expression profiles that closely resemble those of the PHHs, indicating their potential for metabolic activity and liver‐specific functions. Furthermore, employing high‐throughput sequencing technologies, such as RNA sequencing (including single‐cell RNA sequencing), allows for a more nuanced understanding of cellular heterogeneity and gene expression variability within HLC populations, thereby enhancing confidence in their functional capabilities [27].

**TABLE 2:** Gene marker expression levels during human pluripotent stem cell (hPSC) differentiation into hepatocyte‐like cells (HLCs).

Gene type	Genes	ESCs/iPSCs	Definitive endoderm	Hepatic specified endoderm	Hepatic progenitor cells	Early HLCs	Mature HLCs
Pluripotency markers	*OCT3/4*						
	*Nanog*	+	−	−	−	−	−
	*SSEA4*	+	+/−	−	−	−	−
	*TRA‐1*	+	−	−	−	−	−
	(*−1–60*, */−1–81*)	+	−	−	−	−	−
Mesendoderm genes	*Brachyury (T)*	−	+	−	−	−	−
	*MixL1*	−	+	−	−	−	−
Endodermal genes	*FGF17*	−	+	−	−	−	−
	*SOX17*	−	+	+/−	+/−	+/−	−
	*FOXA2*	−	+	+	+	+	+
	*GATA4*	−	+	+	+	+	+
	*CXCR4*	+	+	+	+	+	−
Liver‐enriched TFs	*HNF1α*	−	−	−	+	+	+
	*HNF1β*	−	−	−	+	+	+
	*HNF4α*	−	−	+	+	+	+
	*CEBP*	−	−	+	+	+	+
	*PXR*	−	−	−	−	−	+
	*CAR*	−	−	−	−	−	+
Liver‐related markers	*ALB*	−	−	+/−	+	+	+
	*AFP*	−	−	−	+	+	−
	*TTR*	−	−	−	+	+	+
	*α1AT*	−	−	−	−	+	+
	*ASGPR1*	−	−	−	−	+/−	+
	*TAT*	−	−	−	−	−	+
	*CK8*	−	−	−	+	+	+
	*CK18*	−	−	−	+	+	+
	*G6P*	−	−	−	−	−	+
CYPs	*CYP1A2*	−	−	−	−	−	+
	*CYP2A6*	−	−	−	−	−	+
	*CYP2B6*	−	−	−	−	−	+
	*CYP2C9*	−	−	−	−	−	+
	*CYP2C19*	−	−	−	−	−	+
	*CYP2D6*	−	−	−	−	−	+
	*CYP3A4*	−	−	−	−	−	+
	*CYP2E1*	−	−	−	−	−	+
	*CYP7A1*	−	−	−	−	−	+
	*CYP3A5*	−	−	−	−	+	−
	*CYP3A7*	−	−	−	−	+	−
Metabolic‐associated genes	*MAOA/B*	−	−	−	−	−	+
	*APO*	−	−	+	+	+	+
	*UGT1A1*	−	−	−	−	+	+
	*UGT1A6*	−	−	−	−	+	+
	*UGT1A9*	−	−	−	−	+	+
	*UGT2B7*	−	−	−	−	+	+
	*SULT2A1*	−	−	−	−	−	+
	*SULT1A1*	−	−	−	−	−	+
	*GSTP1*	−	−	−	−	−	+
Biliary epithelial cell markers	*CK19*	−	−	−	+	+/−	−
	*CK7*	−	−	+	+	−	−
	*SOX9*	−	−	−	+	+	−
Canalicular transporters	*MRP2*	−	−	−	−	−	+
	*BSEP*	−	−	−	−	−	+
Basolateral transporters	*NTCP*	−	−	−	−	−	+
	*OATPs*	−	−	−	−	−	+
	*Oct‐01*	−	−	−	−	−	+
	*Oct‐02*	−	−	−	−	−	+
	*MRP6*	−	−	−	−	−	+
	*MRP3*	−	−	−	−	−	+
Liver‐specific miRNAs	*miR‐122*	−	−	−	−	−	+
	*miR‐148*	−	−	−	−	−	+
	*miR‐194*	−	−	−	−	−	+
Proliferation markers	*Ki67*	+	−	−	+	−	−

Abbreviations: AFP, alpha‐fetoprotein; ALB, albumin; APO, apolipoprotein; ASGPR1, asialoglycoprotein receptor 1; BSEP, bile salt export pump; CAR, constitutive androstane receptor; CEBP, CCAAT enhancer‐binding protein beta; CK8, cytokeratin 8; CXCR4, C‐X‐C motif chemokine receptor 4; CYP1A2, cytochrome P450 1A2; FGF17, fibroblast growth factor 17; FOXA2, forkhead box protein A2; G6P, glucose‐6‐phosphatase; GATA4, GATA‐binding protein 4; GSTP1, glutathione S‐transferase Pi 1; HNF4, hepatocyte nuclear factor 4; MAOA/B, monoamine oxidase A/B; miR‐122, microRNA 122; MixL1, mix paired‐like homeobox; MRP2, multidrug resistance protein 2; NTCP, Na^+^‐taurocholate cotransporting polypeptide; OATPs, organic anion‐transporting polypeptides; OCT3/4, octamer‐binding transcription factor 3/4; PXR, pregnane X receptor; SOX17, SRY‐box transcription factor 17; SSEA4, stage‐specific embryonic antigen 4; SULT2A1, sulfotransferase family 2A member 1; TAT, trans‐activator of transcription; TRA‐1, T‐cell receptor alpha locus 1; TTR, transthyretin; UGT1A1, UDP‐glucuronosyltransferase 1A1; α1AT, alpha‐1 antitrypsin.

#### 1.1.4 Proteomic Analysis

Liver protein expression profiling is another approach for evaluating the PHH and HLC phenotypes. HLC proteome analysis provides basic information on hepatocyte maturity and function, and therefore, represents an important quality control method [28]. Protein detection is commonly performed using antibody‐based techniques, such as flow cytometry, immunostaining and western blotting (WB). Mass spectrometry (MS) is used for the large‐scale evaluation of cell proteomes [29, 30, 31]. Proteomic analysis has some limitations, such as accurate identification and quantification of proteins. Additionally, sample preparation and data analysis methods may introduce biases or errors that can affect dataset reliability. Furthermore, the cost‐ and time‐intensive nature of proteomic experiments is a limiting factor for researchers. To overcome these obstacles, various strategies, such as sample fractionation, enrichment and integration with other omics datasets, have been developed [32]. Proteomic analyses of HLCs reveal significant differences compared to PHHs. Western blotting and mass spectrometry (MS) can assess the quality of HLCs by evaluating the expression levels of key markers, including ALB, CYPs and HNF4α. High levels of ALB and CYP expression in HLCs are indicative of mature hepatocytes capable of performing essential liver functions, such as drug metabolism [33]. When comparing HLCs to 24‐h cultured primary human hepatocytes (PHHs), it is crucial to establish baseline reference levels for these proteins. The expression profiles observed in high‐quality HLCs should closely resemble those found in 24‐h cultured PHHs, providing confidence in their functional capabilities. For instance, robust ALB and CYP expression levels similar to those seen in PHHs would suggest successful differentiation and maturation [34, 35]. Furthermore, MS can detect posttranslational modifications that may influence protein activity, further enhancing the understanding of HLC functionality [33].

#### 1.1.5 Flow Cytometry

Flow cytometry is widely used to profile cell surfaces and intracellular protein expression. It is a rapid and reliable approach to quantitatively profile large numbers of cells, and allows users to enrich cell populations for further analysis or subculture. This approach has been used to profile stem cell differentiation into the hepatocyte lineage, as summarised in Table 3.

**TABLE 3:** Marker expression levels during hPSC differentiation into HLCs.

Differentiation stages	Intracellular markers	Surface and transmembrane markers
Pluripotency markers	OCT3/4 and NANOG	SSEA3/4, TRA‐1‐60 and TRA‐1‐81
Definitive endoderm	SOX17 and FOXA2	CXCR4, CD117, EPCAM, CD49e and CD51
Hepatic endoderm	AFP, HNF4α, GATA4, CK7, CK18 and CK19	CD29, CD34, CD49F, C‐Kit, C‐MET, THY1, NCAM, EPCAM, N‐cadherin and E‐cadherin
Hepatocyte maturation	AFP, ALB, A1AT, CYPs, CK8 and CK18	CD29, CD49F, ASGPR1, E‐cadherin and CD81

Abbreviations: A1AT, alpha‐1 anti‐trypsin; AFP, alpha‐fetoprotein; ALB, albumin; ASGPR1, asialoglycoprotein receptor 1; CD117, cluster of differentiation 117; CXCR4, C‐X‐C motif chemokine receptor 4; CYP, cytochrome P450; EPCAM, epithelial cellular adhesion molecule; FOXA2, forkhead box protein A2; GATA4, GATA‐binding protein 4; HNF4α, hepatocyte nuclear factor 4 alpha; NCAM, neural cell adhesion molecule; OCT3/4, octamer‐binding transcription factor 3/4; SOX17, SRY‐box transcription factor 17; SSEA4, stage‐specific embryonic antigen 4; TRA‐1, T‐cell receptor alpha locus 1.

Flow cytometry was initially designed to analyse the cell surface marker expression and other characteristics of nonadherent cells, such as cell size. However, the enzymatic detachment of adherent cells, such as HLCs, may result in the modification or destruction of their surface markers. To avoid the detrimental postdetachment effects, cell suspensions are fixed or rapidly processed to limit the artefacts. Hydrodynamic forces in flow cytometry are used to align the cells. Fluidic stress is detrimental to large cells, such as PHHs or HLCs, which can clog the fluidic channels, thereby compromising cell analysis and recovery. To prevent fluidic stress, a large nozzle size should be used. Necrotic or apoptotic cells also compromise the dataset quality and subcultured cell populations. Therefore, removal of dead/apoptotic cells is critical for efficient flow cytometry [36, 37]. In comparison to 24‐h cultured PHHs, flow cytometry can reveal critical differences in the expression of liver‐specific proteins, such as albumin (ALB), alpha‐fetoprotein (AFP) and cytochrome P450 enzymes (CYPs). Additionally, flow cytometry facilitates the identification of cell populations at various stages of differentiation, which allows for a better understanding of the development process, enabling researchers to monitor the transition from pluripotent stem cells to definitive endoderm and then to mature hepatocytes. By integrating flow cytometry data with other characterisation techniques, researchers can achieve a comprehensive assessment of hepatocyte‐like cell (HLC) quality, ensuring their suitability for applications in drug development and disease modelling [36, 37].

#### 1.1.6 Immunostaining

By first exposing the cells to specific ‘primary antibodies’ recognising the target protein and then to tagged ‘secondary’ antibodies, the user can visualise the presence and localisation of the target marker in situ. Such an analysis does not require the adherent cells to be enzymatically detached from each other or their matrix. Therefore, the cell morphology, cell‐to‐cell communication and cell‐to‐matrix attachment were better maintained. Using this method, several nuclear or cytoplasmic markers can be efficiently detected, and their subcellular localisation can be recorded. Various antibodies, such as ALB and urea cycle proteins, are commercially available for the detection of human proteins and evaluation of the hepatic phenotype (Tables 3 and 4). Immunostaining is limited by the quality of primary and secondary antibodies. Although monoclonal antibodies are preferred, they are not always available. Therefore, proper positive and negative control samples are necessary to draw solid conclusions and accurately confirm the cell identity and maturity [38].

**TABLE 4:** Commonly used markers for immunophenotyping of HLCs.

Protein name	Cellular location	Differentiation state
Oct3/4, NANOG, SOX2	Nucleus	Undifferentiated PSCs
CXCR4	Membrane	Definitive endoderm
SOX17	Nucleus	Definitive endoderm
AFP	Cytoplasm	Hepatic progenitor
ALB	Cytoplasm	Maturation
AAT	Cytoplasm	Maturation
KRT18	Cytoplasm	Maturation
UGTA1	Cytoplasm	Maturation
APOB	Membrane	Maturation
TTR	Cytoplasm	Maturation
HNF4	Nucleus	Maturation
CYPs, e.g., CYP3A4, 1A2	Cytoplasm	Maturation
GLUL	Cytoplasm	Maturation
CPS1	Cytoplasm	Maturation
E‐cadherin	Membrane	Maturation of cell polarity in HLC
ZO1	Membrane	Maturation of cell polarity in HLC
NTCP	Canalicular (apical) domain of hepatocytes	Maturation of apical–basolateral polarity and bile canaliculi
BSEP	Canalicular (apical) domain of hepatocytes	Maturation of apical–basolateral polarity and bile canaliculi
ANO6	Canalicular (apical) domain of hepatocytes	Maturation of apical–basolateral polarity and bile canaliculi

Abbreviations: AAT, alpha‐1 anti‐trypsin; AFP, alpha‐fetoprotein; ANO6, anoctamin 6; APOB, apolipoprotein B; BSEP, bile salt export pump; CPS1, carbamoyl‐phosphate synthase 1; CYP, cytochrome P450; GLUL, glutamate–ammonia ligase; HNF4, hepatocyte nuclear factor 4; KRT18, keratin 18; NTCP, Na^+^‐taurocholate cotransporting polypeptide; OCT3/4, octamer‐binding transcription factor 3/4; SOX2, SRY‐box transcription factor 2; SSEA4, CXCR4, C‐X‐C motif chemokine receptor 4; TTR, transthyretin; UGTA1, UDP‐glucuronosyltransferase A1; ZO1, zonula occludens‐1.

#### 1.1.7 Western Blotting

Western Blotting is a laboratory method used to identify proteins extracted from lysed cells and tissues. Such investigations are composed of three steps: (1) separation of the proteins according to their size, (2) transfer of the separated proteins to a solid support, such as nylon or polyvinylidene difluoride and (3) targeting and visualising the proteins of interest using specific antisera and reagents [39]. WB also allows for the detection and discrimination of posttranslated and truncated protein forms. The choice of detection method depends on the specific requirements and equipment available in the laboratory. When working with tissue lysates or tissue culture supernatants containing serum and endogenous immunoglobulins, it is important to select a primary antibody raised in a different species from that of the sample to ensure the sensitivity and specificity [38]. However, efficiency of WB is limited by the quality of primary and secondary antibodies.

#### 1.1.8 Secretory Assays

Liver cells produce, metabolise and secrete various soluble products in the human body. In this section, we discuss laboratory technologies aimed at measuring secreted proteins and metabolites in the supernatants of differentiated HLCs and PHHs.

### 1.2 Enzyme‐Linked Immunosorbent Assay (ELISA)

Enzyme‐Linked Immunosorbent Assay is a common laboratory assay that was first described more than 50 years ago [40]. The secretion of many proteins, including ALB, A1AT and fibrinogen, in hepatocytes has been analysed and quantified using ELISA. Such an analysis can be performed immediately using fresh conditioned media or the samples can be archived for future analysis [41, 42]. Similar to immunostaining and WB, ELISA kits are limited by the selectivity and sensitivity of the antibodies used.

### 1.3 Colorimetric Assay

The liver is responsible for maintaining the nitrogen balance in the body. Nitrogen is used in the synthesis of proteins, pyrimidines, purines and carbohydrates, and excess nitrogen is excreted in the form of urea. Ammonia metabolism and urea synthesis are measured by using colorimetric biochemical assays [41, 43]. Urea is an unstable molecule; therefore, care is required when preparing, storing and measuring its samples.

### 1.4 Liquid Chromatography (LC)‐Tandem MS (LC–MS/MS)

Drug safety depends on the purity, specificity and toxicity of active substances and their metabolites. During manufacturing, the quality and quantity of a drug are typically determined using different analytical methods, such as titrimetric, chromatographic, spectroscopic and electrochemical methods. Among the chromatographic techniques, LC plays an important role in the pharmaceutical industry. Since its application in 1980 for the assessment of bulk drug materials, LC has become a principal method in both the United States and the European Pharmacopoeia [43, 44]. Pharmacological response is generally related to the concentration of the drug at the receptor site. However, drug concentration cannot be readily measured directly at the site of action; therefore, the majority of bioavailability studies measure drug and/or metabolite concentrations in biological fluids, such as blood, plasma, urine [45] and cell lysates [46, 47, 48]. LC–MS/MS combines high‐performance LC (HPLC) with MS. This technique is commonly used in laboratories for qualitative and quantitative analyses of drug substances, drug products and biological samples throughout all phases of drug development in research and quality control. LC–MS/MS plays crucial roles in the evaluation and clarification of the bioavailability, bioequivalence and pharmacokinetics of medicinal components [49]. MS‐based metabolomics is increasingly used for drug discovery owing to its high‐resolution, high‐throughput qualitative and quantitative sensitivity and widespread availability [50]. In hepatocytes, drugs are metabolised through modifications, such as oxygenation, *N*‐demethylation, glucuronidation, sulfation and glutathione (GSH) conjugation. LC–MS/MS is helpful in understanding drug processing, mechanisms of action and off‐target effects [51]. The metabolism of acetaminophen, diclofenac, lamotrigine, midazolam, propranolol and salbutamol has been analysed in PHHs and HLCs using LC/MS/MS to identify key metabolites following CYP‐mediated biotransformation [52]. In another study, bile acid serum samples were analysed using LC–MS/MS [53]. Although it is possible to measure the correct concentrations of drugs and metabolites using LC–MS/MS, a high level of skill is required to generate reliable datasets for downstream analysis [45]. In addition to its application to the analysis of secretory factors, MS has long been used in proteomics. Traditionally, MS has been instrumental in protein research as it facilitates sequence identification and quantification of protein expression. However, in recent years, MS applications have expanded to become an invaluable tool in structural biology. One notable advancement is the emergence of intact protein structure analysis, which is made possible through techniques, such as native MS, top‐down proteomics and ion mobility MS. These methods have revolutionised our ability to probe the intricate details of protein structures, allowing researchers to explore protein–protein and protein–ligand interactions as well as regions of conformational changes. Moreover, MS can be combined with LC to analyse complex samples [54, 55]. LC–MS/MS is a powerful analytical technique but has certain limitations. One of the main limitations of LC–MS/MS is the occurrence of matrix effects, which can lead to ion suppression or enhancement and affect the accuracy. Matrix effects are caused by the presence of co‐eluting matrix components that interfere with the ionisation and fragmentation of the analyte of interest. Matrix effects can be particularly problematic in biological samples, such as blood, plasma or urine, which contain high concentrations of endogenous compounds that can interfere with LC–MS/MS analysis. Matrix effects can also vary between samples, making it difficult to establish a standard calibration curve and leading to inaccurate quantification. Another limitation of LC–MS/MS is the complexity of sample preparation. LC–MS/MS requires a high degree of sample cleanup and preparation to remove interfering compounds, such as proteins, salts and lipids. These impurities induce ion suppression and enhancement. Sample preparation can be time‐consuming and labour‐intensive and can affect the recovery and reproducibility of the analysis. In addition, the use of different sample preparation methods and protocols can lead to variations in the results and limit the comparability of data between laboratories. Therefore, the development of standardised sample preparation protocols is crucial for improving the accuracy and reproducibility of LC–MS/MS analysis [56, 57, 58].

### 1.5 Application of Characterisation Techniques in Assessing HLC Quality

The assessment of HLC quality is critical for their effective applications in research and therapy. Various characterisation techniques are employed to distinguish between high‐quality and low‐quality HLCs, each offering unique insights into cellular properties, functionality and overall suitability for clinical use. For instance, flow cytometry allows for the quantitative measurement of specific cell surface markers and intracellular proteins that are indicative of hepatocyte identity and function. High‐quality HLCs typically express markers such as albumin. Flow cytometry can effectively distinguish between HLCs that exhibit robust expression of these markers and those that do not, thereby assessing their hepatocytic maturation [36, 37]. In addition, qPCR and RNA sequencing are utilised to evaluate the expression levels of liver‐specific genes. The presence of genes like HNF4A, ALB and CYP3A4 in high quantities is indicative of mature HLCs. Low expression levels or the absence of these genes can suggest poor quality or incomplete reprogramming [22]. Moreover, functional assays such as urea production, albumin secretion and drug metabolism studies enable the assessment of the metabolic capabilities of HLCs. High‐quality HLCs demonstrate significant metabolic activity comparable to primary hepatocytes. For instance, measuring urea production can indicate the functionality of the urea cycle, while albumin secretion rates can reflect the synthetic capacity of the cells [59]. In immunofluorescence microscopy, high‐quality HLCs will show strong positive staining for hepatic markers such as cytokeratin 18 and HNF4A. The intensity and specificity of staining can help differentiate well‐differentiated cells from poorly differentiated ones [3]. Additionally, a comprehensive proteomic analysis using mass spectrometry helps identify liver‐specific proteins and metabolic enzymes, providing insights into the functional maturation of HLCs. High‐quality cells will exhibit a proteomic profile that closely resembles that of primary hepatocytes [33]. Furthermore, techniques such as mass spectrometry can assess the metabolic activity of HLCs. High‐quality HLCs should exhibit metabolic profiles consistent with hepatic function, including appropriate levels of metabolites involved in liver metabolism.

### 1.6 Functional Assessments

#### 1.6.1 CYP Inducibility and Activity

Cytochrome P450 enzyme activity and induction assays are important functional evaluations for PHHs and HLCs. CYPs are enzymes that are crucial for the metabolism of various drugs and xenobiotics. Major factors influence CYP activity and function, including genetic polymorphisms, epigenetic factors and nongenetic host factors, such as sex, age and comorbidities [60].

**FIGURE 3:**
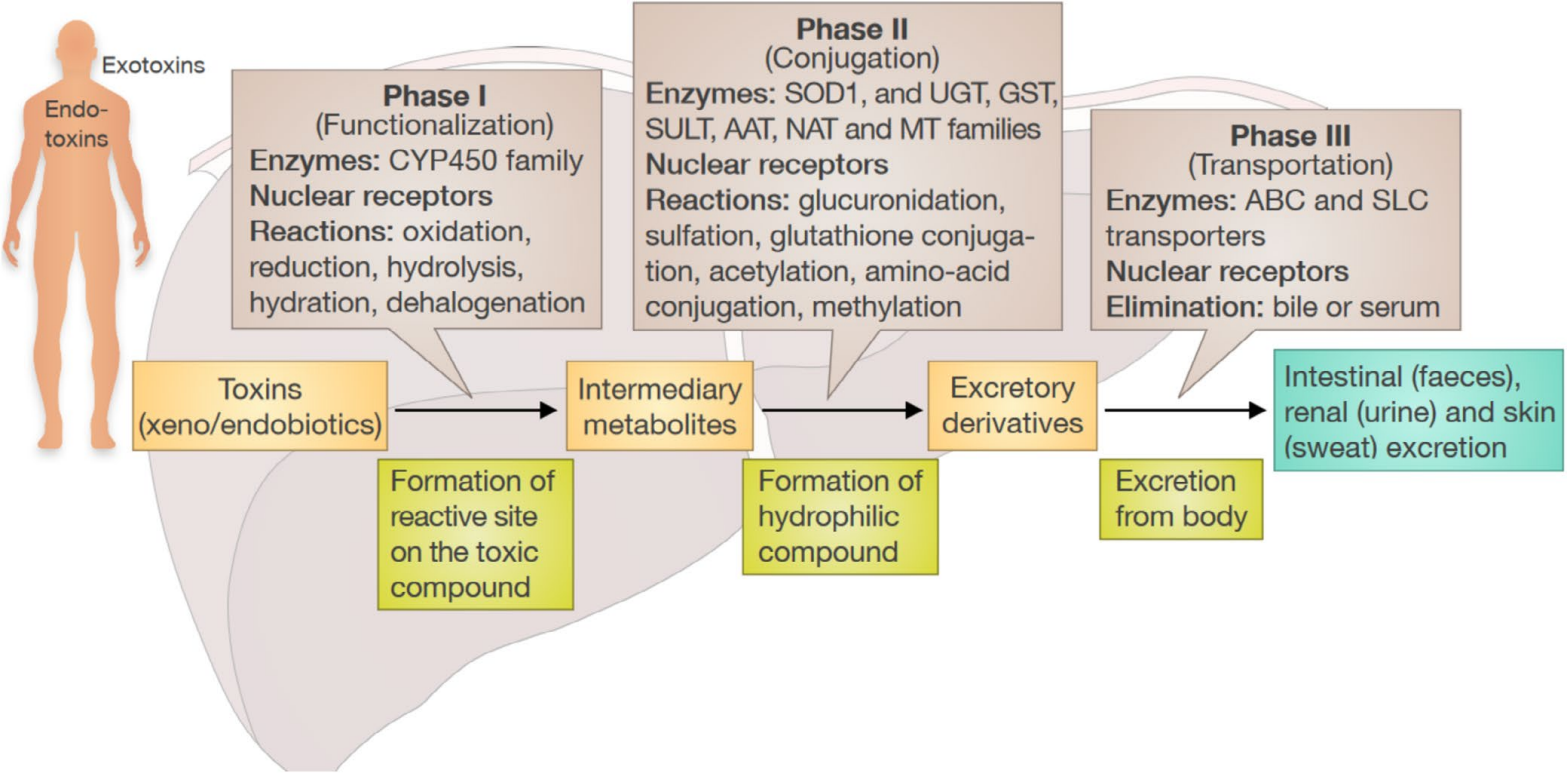
Metabolic pathways. Cytochrome P450 enzymes (CYPs) play a key role in phase I detoxification by making substances more water‐soluble or preparing them for phase II reactions. In phase II detoxification, these intermediates are converted into water‐soluble compounds via conjugation reactions. In phase III detoxification, these compounds are removed from cells using specific transporters. AAT, amino acid transferase; ABC, ATP‐binding cassette; GST, glutathione S‐transferase; MT, methyltransferases; NAT, N‐acetyltransferase; SLC, solute carrier; SOD, superoxide dismutase; SULT, sulfotransferases; UGT, UDP‐glycosyltransferase.

Although over 50 different CYP enzymes have been identified to date, only six are involved in the metabolism of approximately 90% of prescription medications, with CYP3A4 and CYP2D6 being the two most expressed enzymes [61]. Drug metabolism occurs at many sites in the body, including the liver, gut epithelium, lungs, kidneys and blood plasma [61]. As the main site of drug metabolism, the liver plays an important role in detoxifying and facilitating the excretion of xenobiotics, such as the enzymatic conversion of lipid‐soluble compounds to more water‐soluble components [62]. CYPs, which are primarily involved in phase I detoxification, utilise oxygen and nicotinamide adenine dinucleotide (NADH) as cofactors to catalyse reactions aimed at increasing the water solubility of substrates or preparing them for phase II reactions. This transformation may lead to the formation of reactive intermediates, such as epoxides, which possess higher reactivity and potential toxicity than their primary compounds. Additionally, during these reactions, byproducts, such as hydroxyl radicals, may be produced, which are highly reactive and toxic. In phase II detoxification, xenobiotics undergo conjugation reactions, including glucuronidation, glutathione conjugation and sulfation, which convert these reactive intermediate molecules into water‐ and fat‐soluble compounds. Finally, phase III detoxification involves the removal of toxins and metabolic products from cells, facilitated by transporters, such as ATP‐binding cassette (ABC) transporters and nuclear receptors. Notably, the detoxification process is not always linear. In some cases, parent compounds may undergo phase II reactions without prior phase I metabolism, depending on the specific xenobiotic and enzymatic pathways involved [63] (Figure 3).

Klingenberg et al. discovered CYPs while investigating steroid hormone metabolism [64]. The major human CYPs involved in drug metabolism are CYP3A4/5, CYP2E1, CYP1A2, CYP2C9, CYP2D6, CYP2B6 and CYP2C19, which account for approximately 20%–30%, 15%–25%, 10%–25%, 10%–20%, 1.5%–5%, 1%–5% and 1%–4% of total CYPs in the liver respectively [65].

##### 1.6.1.1 CYP 1 Enzyme Family

1A1 and 1A2. The catalytic activities of CYP1 enzymes include hydroxylation and other oxidative alterations of polycyclic aromatic hydrocarbons and aromatic substances. The major family members are listed in Table 5 [60]. Inducers and inhibitors of these enzymes were also mentioned [66, 67]. The common substrates of the CYP1 family are listed in Table 5 [68, 69].

**TABLE 5:** Inducers, inhibitors and substrates of cytochrome P450 enzymes in human hepatocytes [60, 70].

CYPs	Inducers	Inhibitors	Substrates
1A2	Sulfinpyrazone, Ritonavir, Rifampicin, Primaquine, Polychlorinated biphenyls, Polycyclic aromatic hydrocarbon, Phenytoin, Phenobarbital and other barbiturates, Omeprazole, Nelfinavir, Cruciferous vegetables (e.g., broccoli), Coffee, Carbamazepine, Bilirubin, Antipyrine and Aminoglutethimide	Tolfenamic acid, Oral contraceptives, Moricizine, Mexiletine, Furafylline, Fluvoxamine, Enoxacin, Disulfiram, Ciprofloxacin, Cimetidine and α‐naphthoflavone	Caffeine, Melatonin, Duloxetine, Ramelteon, Warfarin, Alosetron, Tacrine and Tizanidine
2A6	Rifampicin, Phenobarbital, Oestrogens, Dexamethasone, Carbamazepine and Artemisinin	Tranylcypromine, Selegiline, Pilocarpine, 8‐methoxypsoralen, (R)‐(+) menthofuran and Decursinol angelate	Coumarin, Nicotine, Quinoline, Valproic acid and Paracetamol
2B6	Vitamin D, Statins (e.g., atorvastatin), Ritonavir, Rifampicin, Phenytoin, Phenobarbital, Nevirapine, Nelfinavir, Metamizole, Hyperforin, 17‐α‐ethinylestradiol, Efavirenz, N,N‐diethyl‐m‐toluamide (DEET), Cyclophosphamide, Carbamazepine, Baicalin and Artemisinin‐type antimalarials	Voriconazole, Ticlopidine, thioTEPA, Sertraline, Raloxifene, 2‐phenyl‐2‐(1‐piperidinyl) propane, Mifepristone (RU486), Imidazoles, Clotrimazole, Clopidogrel and Bergamottin	Nicotine, Bupropion, Cyclophosphamide and Efavirenz
2C8	Statins (e.g., atorvastatin), Ritonavir, Phenytoin, Phenobarbital, Paclitaxel, Nelfinavir, Lithocholic acid, Imatinib, Hyperforin, Fibrates (e.g., gemfibrozil), Dexamethasone and Cyclophosphamide	Trimethoprim, Montelukast and Gemfibrozil	Ibuprofen, Paclitaxel and Cerivastatin
2C9	Statins (e.g., atorvastatin), Ritonavir, Rifampicin, Prednisone, Phenobarbital, Norethindrone, Nifedipine, Nelfinavir, Hyperforin, Glutethimide, Dexamethasone, Cyclophosphamide, Carbamazepine, Bosentan, Barbiturates, Avasimibe and Aprepitant	Voriconazole, Tienylic acid, Sulphaphenazole, Naringenin, Fluconazole and Amiodarone	Celecoxib, Warfarin, Phenytoin, Linoleic acid, Rosiglitazone and Tolbutamide
2C19	Ritonavir, Rifampicin, Nelfinavir, Hyperforin, Efavirenz, Dexamethasone, Carbamazepine, Barbiturates, Baicalin, Artemisinin‐type antimalarials, Antipyrine and Acetylsalicylic acid	Voriconazole, Ticlopidine, Omeprazole, (+)‐N‐3‐benzyl‐nirvanol, Naringenin, Fluvoxamine, Fluoxetine, Clopidogrel and (−)‐N‐3‐benzyl‐phenobarbital	Omeprazole, Amitriptyline, S‐mephenytoin, Clobazam, Lansoprazole, Diazepam and Phenobarbital
2D6	No significant induction by prototypical cytochrome P450 inducer	Quinidine, Paroxetine, Methadone, Haloperidol, Fluoxetine, Flecainide and Bupropion	Perphenazine, Tolterodine, Debrisoquine, Codeine, Timolol, Flecainide, Dextromethorphan, Atomoxetine, Venlafaxine, Desipramine, Metoprolol and Nebivolol
2E1	Pyrazole, Isoniazid, Ethanol and Acetone	Orphenadrine, 4‐methylpyrazole, Disulfiram, Diethyldithiocarbamate and Clomethiazole	Ethanol, Paracetamol, Halothane and Toluene
3A4	Phenytoin, Phenylbutazone, Phenobarbital, Oxcarbazepine, Nevirapine, Nafcillin, Moricizine, Mitotane, Miconazole, Imatinib, Hyperforin, Glucocorticoids, *Ginkgo biloba* , Etravirine, Efavirenz, Dexamethasone, Carbamazepine, Bosentan, Barbiturates, Baicalin, Avasimibe, Artemisinin‐type antimalarials, Aprepitant, Amprenavir, Vinblastine, Valproic acid, Troglitazone, Topiramate, Sulfinpyrazone, Statins, Ritonavir, Rifapentine, Rifampicin and Rifabutin	Voriconazole, Verapamil, Troleandomycin, Ritonavir, Nicardipine, Naringenin, Mifepristone, Mibefradil, Ketoconazole, Irinotecan, Isoniazid, Grapefruit juice, Ethinylestradiol, Erythromycin, Diltiazem, Clarithromycin and Azamulin	Testosterone, Eletriptan, Eplerenone, Alfentanil, Aprepitant, Darifenacin, Darunavir, Quetiapine, Lopinavir, Lurasidone, Conivaptan, Saquinavir, Sildenafil, Lovastatin, Maraviroc, Midazolam, Nisoldipine, Simvastatin, Tipranavir, Tolvaptan, Sirolimus, Ticagrelor, Triazolam, Vardenafil, Dasatinib, Felodipine, Fluticasone, Everolimus, Budesonide, Buspirone, Dronedarone and Indinavir
3A5	Glucocorticoids, Rifampicin, Carbamazepine, Phenobarbital and Phenytoin	Erythromycin, Ketoconazole, Clarithromycin and Verapamil	Midazolam, Nifedipine and Testosterone
3A7	Glucocorticoids	Halometasone	Dehydroepiandrosterone (DHEA) and Retinoic acid

##### 1.6.1.2 CYP 2 Enzyme Family

2A6, 2B6, 2C8, 2C9 and 2C19. CYP2A6 is the main enzyme involved in the oxidative conversion of nicotine to inactive cotinine. The inducers, inhibitors and substrates of the enzymes are listed in Table 5 [71, 72].

##### 1.6.1.3 CYP 3 Enzyme Family

CYP3A4, CYP3A5 and CYP3A7. Among the members of this family, CYP3A4 is a major player in human liver metabolism. Owing to its large and flexible active site CYP3A4, it can metabolise many components, especially lipophilic compounds with large structures, such as immunosuppressants and anticancer drugs. CYP3A5 and CYP3A7 are other members of this family that are usually expressed during the foetal period and are downregulated after birth. Inducers, inhibitors and the common substrates of these enzymes are included in Table 5 [73, 74].

Use of drugs that interact with each other can lead to alterations in enzyme activity, resulting in compromised therapeutic effects and increased risk of adverse drug reactions. This highlights the importance of understanding and modelling human drug metabolism prior to clinical trials [75, 76].

**FIGURE 4:**
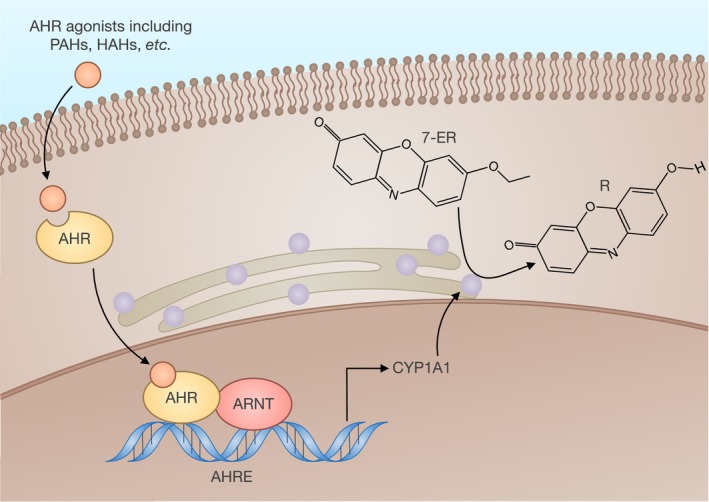
Schematic illustration of aryl hydrocarbon receptor (AHR) activation and conversion of 7‐ethoxyresorufin to resorufin by CYP1A1. AHR, aryl hydrocarbon receptor; AHRE, aryl hydrocarbon response element; ARNT, AHR nuclear translocator; HAHs, halogenated aromatic hydrocarbons; PAHs, nonhalogenated polycyclic aromatic hydrocarbons.

Many assays, including the P450‐Glo (Promega) and ethoxyresorufin‐O‐deethylase (EROD) assays, are used to estimate CYP activity in hepatocyte preparations. The EROD assay involves the oxidative de‐ethylation of 7‐ethoxyresorufin (7‐ER) to resorufin, catalysed by CYP1A1. This enzyme is the principal P450 isozyme involved in O‐deathylation (Figure 4). The reaction is carried out at high concentrations of 7‐ER so that enzyme catalysis is nearly maximal [77].

Many factors need to be considered for EROD assay. First, the maximum EROD activity varies from one CYP1A1 inducer to another. Second, EROD activity does not follow a conventional saturation curve as the inducer concentration increases but reaches a maximum and then declines [78].

### 1.7 Drug–Drug Interactions

Cytochrome P450 enzyme inhibition and induction are key mechanisms in drug–drug interactions, which can broadly be defined as the effects of one drug on the metabolic clearance of another. Thus, the accurate prediction of human drug metabolism and interactions using preclinical models is important for safe drug dosing in the clinic [79, 80, 81]. In addition to their role in detoxification, CYPs are central players in prodrug–drug activation. This activation step is crucial for the therapeutic efficacy of these drugs. Furthermore, variations in individual CYP enzyme activity can have a profound impact on drug responses and may underlie inter‐individual differences in drug efficacy and side effects. Inducibility assays are widely used to characterise PHHs and HLCs. HLCs derived from PSCs or other sources are used to study drug metabolism and liver‐specific functions. These cells are exposed to specific inducers, such as phenobarbital, isoniazid and omeprazole, to assess the metabolism of the compounds and the potential for off‐target effects [82, 83]. Although HLCs are valuable in mimicking liver‐related functions, they may not fully replicate the complexity of PHHs in terms of their metabolic activity and drug metabolism. Additionally, the choice of inducers may affect the relevance of the results, as some inducers do not accurately mimic the physiological conditions in the liver. Researchers must be cautious when extrapolating their findings from HLC assays to in vivo biology and should consider conducting additional experiments with freshly isolated PHHs or animal models to test their hypotheses [83].

### 1.8 Drug Safety

The liver is the major site of metabolism and drug biotransformation; thus, PHHs have been used as in vitro tools for toxicological and pharmacological testing. Owing to the limitations associated with PHHs, as stated earlier, PSC‐derived HLCs have been used as an alternative model to predict drug‐induced cytotoxicity (Table 6) [84]. To assess the specificity and sensitivity, in vitro‐generated HLCs were treated with various components that cause hepatotoxicity at different doses. Cell viability assays, such as MTT, MTS and Orangu kit assays, have been used to evaluate cell health. Since HLCs can be maintained in culture for extended periods, assessment of both acute and chronic toxicity is feasible [85, 86].

**TABLE 6:** Common drugs used to test for primary human hepatocyte (PHH) and HLC sensitivity and specificity [87, 88].

Hepatotoxicity	Compounds
Toxic	Acetaminophen, Amiodarone, Benzbromarone, Clozapine, Diclofenac, Flurbiprofen, Mebendazole, Mefenamic acid, Phenacetin, Phenylbutazone, Quinine, Trazodone HCl, Troglitazone, Acetazolamide, Betahistine 2HCl, Captopril, Chloramphenicol palmitate, Ciprofloxacin HCl, Clomiphene citrate, Clomipramine, Cyclophosphamide, Cyproterone acetate, Danazol, Dapsone, Estrone, Hydroxyurea, Imipramine HCl, Isoniazid, Maleic acid, Methimazole, Nifedipine, Norgestrel, Nortriptyline HCl, Piroxicam, Progesterone, Pyrazinamide and Tamoxifen
Nontoxic	Aspirin, Buspirone, Dexamethasone, Dextromethorphan HBr, Fluoxetine, Miconazole, Prednisone, Propranolol, Rosiglitazone and Warfarin

*Note:* Solvent dimethyl sulfoxide (DMSO) concentration varies between 0.1% and 1% due to solubility issues [89] Increasing the level of DMSO in the culture medium leads to a reduction in CYP3A4 activity [90].

#### 1.8.1 Mitochondria and Cellular Bioenergetics

Mitochondria play important roles in energy production and nitrogen balance in hepatocytes. The urea cycle comprises five enzymes and two critical mediators (OTC and CPS‐1) located within the cellular mitochondria. Mitochondrial activity can be assessed based on gene expression, replication, ultrastructure and respiration (oxygen consumption). mtDNA replication is regulated by nuclear‐encoded mitochondrial transcription factor A (TFAM) and mitochondria‐specific DNA polymerase gamma (POLG), which have catalytic (POLG1) and accessory (POLG2) subunits. The OCR has been widely used to measure ATP production via oxidative phosphorylation in mitochondria, the measurement of the oxygen consumption ratio (OCR) has been widely described [87, 88]. One of the more common assays to measure OCR in living cells is the Cell Mito Stress test using the XFe96 extracellular flux analyser (Seahorse, Boston, MA, US). This method requires a small number of cells; however, accurate cell counting and cellular homogeneity are important to minimise the variability between groups [91].

#### 1.8.2 Lipid Uptake and Metabolism

**FIGURE 5:**
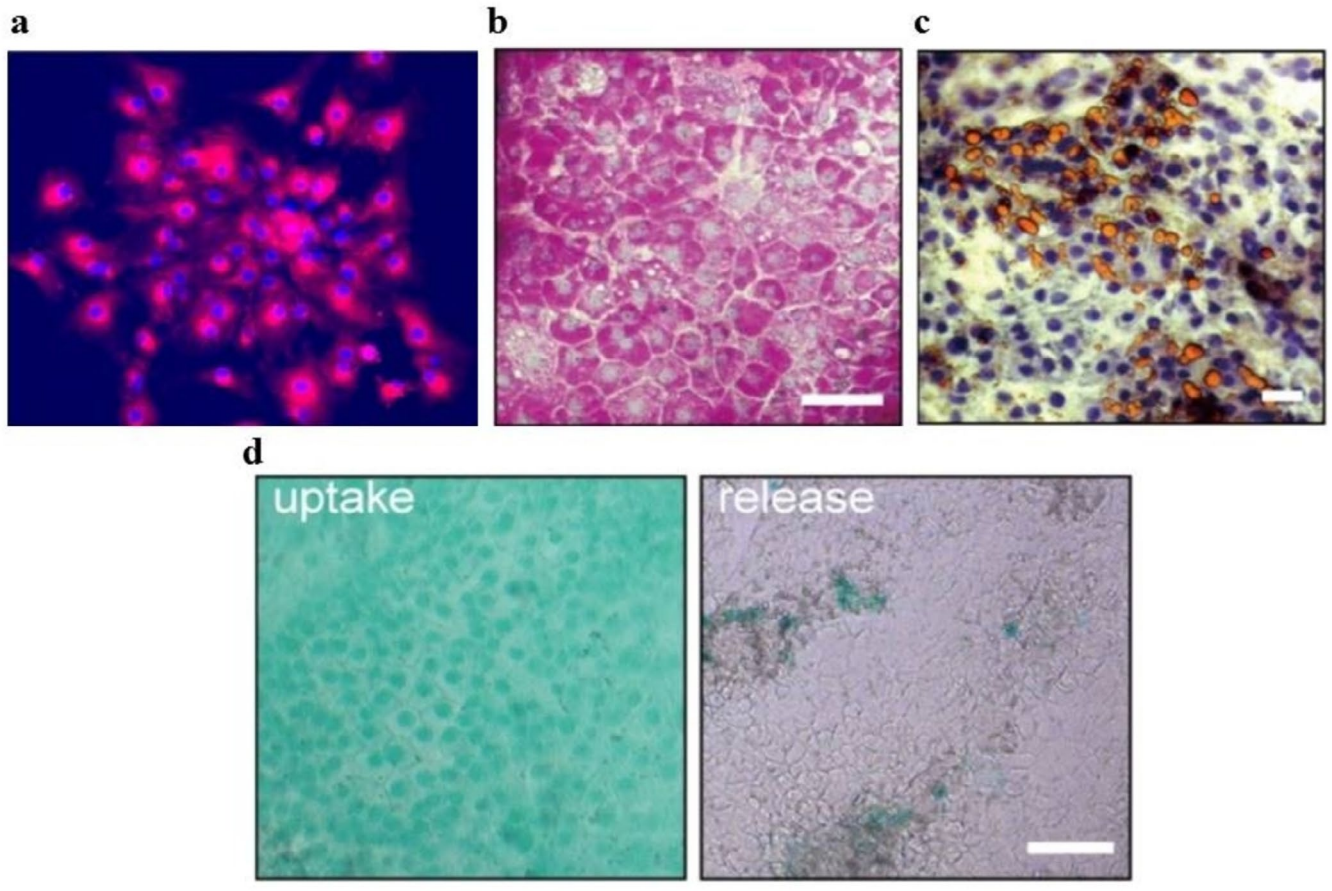
Low‐density lipoprotein (LDL) uptake, periodic acid‐Schiff (PAS) and oil red O (ORO) staining and indocyanine green (ICG) uptake. (a) LDL uptake by umbilical cord vein mesenchymal stem cell–derived hepatocytes. DiI‐Ac‐LDL and DAPI are shown in red and blue respectively (200× magnification) [95]. (b) HLCs in hepatobiliary organoids (HBOs) storing glycogen were evaluated via PAS staining. (c) ORO staining revealed the accumulation of liquid in HLCs. (d) ICG uptake and release were observed in HBO HLCs. All assays were conducted on day 45. Scale bars: 50 μm [96]. ICG, indocyanine green; LDL, low‐density lipoprotein; ORO, oil red O; PAS, periodic acid‐Schiff.

Low‐density lipoprotein (LDL) uptake is another functional assay frequently performed on PHHs and HLCs. LDL particles transport two types of lipids, cholesterol and triglycerides. LDL is derived from very low‐density lipoprotein (VLDL) produced by the liver, along with apoprotein B‐100. Endothelial lipase converts VLDL to LDL. Extra LDL is taken up by hepatocytes via the LDL receptor (LDL‐R) and converted to cholesterol for bile acid and de novo lipoprotein production [92]. The LDL assay requires fluorescently labelled LDL supplementation in the cell culture medium. LDL uptake is a relatively inexpensive and easy test to conduct, with visualisation by fluorescence microscopy (Figure 5a), and can be coupled with cell sorting for analysis and cell enrichment [92, 93]. Although useful, LDL uptake is not liver‐specific and efficiently labels both vascular endothelial cells and macrophages/Kupffer cells [94].

#### 1.8.3 Glycogen Storage

Periodic acid‐Schiff (PAS) staining is used to detect carbohydrates in hepatocytes. This technique was first used to detect mucin by McManus in 1946 [97]. PAS staining can highlight the carbohydrate‐containing molecules, such as glycogen, in skeletal muscle, cardiac tissues, kidneys and liver cells [98]. PAS staining is not a specific assay to assess the hepatic metabolic activity; however, it demonstrates the ability of hepatocytes to synthesise and store glycogen. PAS technique is based on the reactivity of the free aldehyde groups of carbohydrates with the Schiff reagent to form a deep purplish red magenta product in the cytoplasm (Figure 5b), with the nuclei counterstained using haematoxylin [39]. When PSCs are induced to differentiate into HLCs, the population of differentiating cells can be evaluated as the percentage of PAS‐positive cells [99, 100]. However, one limitation of PAS staining is that it is not specific to glycogen unless used in combination with diastase treatment.

#### 1.8.4 Oil Red O (ORO) Staining

Oil Red O is a fat‐soluble diazo dye used to detect the neutral lipids in frozen fixed tissues [101] or live cells [102] Accumulation of lipid droplets is identified using ORO staining, indicating the ability of HLCs to metabolise lipids [46] (Figure 5c). ORO staining is a relatively easy and quick procedure; however, it is not liver‐specific and cannot stain all lipids [101, 103].

#### 1.8.5 Indocyanine Green (ICG) Uptake and Release

Indocyanine Green uptake and release are other functional tests used to assess the HLC identity and hepatic maturation [104, 105]. ICG (C_43_H_47_N_2_NaO_6_S_2_) is a water‐soluble anionic compound with an affinity for plasma proteins [106]. ICG trafficking has been largely described in in vitro analyses of primary hepatocytes and cell lines [107]. Hepatic cells take up ICG via transporter organic anion transport proteins within 30 min of exposure [108, 109]. Once internalised, ICG was visible as a green dye in the cytoplasm of the treated cells by microscopy (Figure 5d). The excretion of an unchanged dye requires 1–2 h, via the ATP‐dependent multidrug resistance protein 2 transporter (ABCC2I) [110], offering a rapid and efficient method to evaluate phase I–III metabolic activities [107]. ICG uptake/release is a relatively simple assay that can be conducted using commonly available laboratory equipment without the need for specialised training [111]. However, this assay had certain limitations that must be considered. ICG uptake can be influenced by factors, such as the expression of uptake transporters, which differ between 2D and 3D cultures. Therefore, the assay may not always accurately reflect the true function of the liver, particularly in cases where the liver function is compromised. Another limitation of the ICG uptake and release assay is that it only provides information on liver function at a single time point. Therefore, it may not be a reliable indicator of changes in liver function over time or in response to treatment [112]. In 3D cultures, this assay is limited by ICG diffusion, leading to reduced uptake. In addition, ICG may bind to the extracellular matrix in 3D cultures, further limiting its uptake by hepatocytes [113].

#### 1.8.6 Hepatocyte Transplantation In Vivo

Successful transplantation of functional HLCs into acute or chronic liver failure models is crucial for their future clinical use. Transplanted cells support injured animals by compensating for the compromised liver function [114]. Several preclinical models have been developed and validated. By implanting HLCs into different animal models, researchers have validated the maturation level and functionality of these cells. The establishment of common liver injury models can be classified as noninvasive, invasive and genetic models. Noninvasive models include oral administration of agents that induce hepatotoxicity, including chemically induced, drug‐induced, radiation‐induced, metal‐induced (e.g., mercury) and diet‐induced (e.g., alcohol and high‐fat diet) options. Noninvasive models are often preferred due to their ease of use and low cost; however, they may not accurately reflect human conditions. Surgical methods, such as portal vein and bile duct ligation, in addition to injections, are employed in invasive models to induce liver injury. These models provide more relevant liver injury phenotypes; however, they are complex and require specialised expertise. Genetic models encompass transgenic or knockout animals with specific genetic modifications used to study hepatotoxicity. Genetic models offer the advantage of studying specific genes or pathways involved in hepatotoxicity but may not fully represent the complexity of human liver diseases [115, 116]. Overall, the type of animal model depends on the specific research question and desired level of complexity and accuracy. Transplanted liver cells or HLCs are morphologically indistinguishable from native hepatocytes, despite some studies reporting the larger size of murine cells compared to that of implanted human hepatocytes or HLCs [117]. Furthermore, the common cell dose injected into rodents only reaches 3%–5% of the total parenchymal cells. Donor cells or cellular components can be detected in liver biopsies using high‐resolution molecular techniques. Previous studies have provided direct evidence of cell engraftment using human‐specific antibodies and/or primers, sex chromosomes and HLA antigens mismatches [118, 119, 120]. However, liver biopsy is invasive and cannot be frequently repeated. Additionally, biopsies have a significant risk of sampling errors, especially when donor cells constitute a small proportion of the resident hepatocytes. Therefore, other technologies, such as positron emission tomography and fluorescence‐based imaging, have been developed to noninvasively track the engrafted cells. Consistent functioning of donor cells is an indirect but efficient marker of cell engraftment and survival, particularly in preclinical models with one or more altered/missing hepatic metabolic activities. Alanine transaminase and aspartate transaminase are conventional biomarkers for hepatic cell injuries. Any decline in their serum levels reflects an improvement in the liver function. However, these serological markers do not provide direct information regarding the number of successfully engrafted cells or site of integration.

### 1.9 Common Cell Transplantation Routes

**FIGURE 6:**
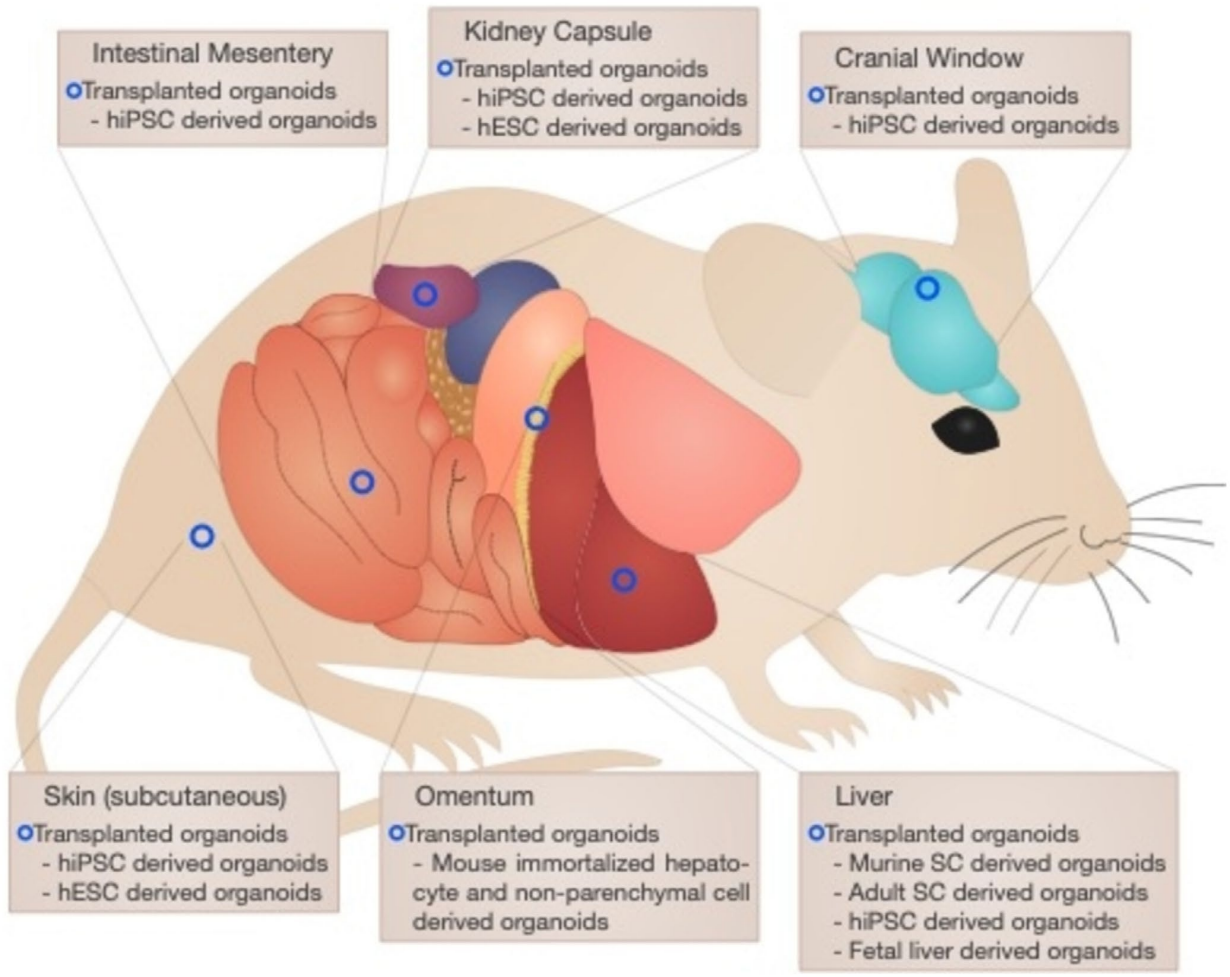
Different transplantation routes used in mouse models. This figure illustrates different transplantation routes, including the cranial window, kidney capsule, intestinal mesentery, liver, omentum and under the skin routes, for cell infusion and implantation.

Many sites, including the liver [121, 122], intestinal mesentery, cranial window, intrasplenic, under the kidney capsule, subcutaneous, omentum, spleen and lymph nodes, are used for hepatocyte transplantation [10] (Figure 6). Each route has its unique advantages and disadvantages. The spleen is a common site for hepatocyte transplantation as it provides a supportive space for transplantation and can be accessed percutaneously without a major surgical incision. As the spleen drains the fluid into the liver via the portal vein, intrasplenic transplantation can be used to effectively seed the liver [123]. Hepatocyte transplantation into the spleen may affect its normal function [124]. Another common transplantation route is the subcapsular space of the kidneys [125], which provides a large pouch for transplanted cells and facilitates long‐term engraftment. Although transplantation under the kidney capsule is an acceptable model for functional testing, it is not clinically viable [126]. The subcutaneous space is a clinically relevant transplantation site that accommodates many cells and rescues the failing liver function in rodents [114, 127]. PHHs and HLCs are also transplanted into the intestinal mesentery, which has a rich blood supply that supports the transplanted cell survival and function. Mesentery provides a large surface area for cell engraftment, enabling efficient integration into the host tissue. However, one drawback is the risk of immune rejection owing to the proximity of the transplanted cells to the gut‐associated lymphoid tissue [128]. Transplantation through the cranial window facilitates the direct visualisation of transplanted cells, allowing real‐time monitoring of their engraftment and function. This route also provides easy access for repeated sampling and analyses. However, its invasive nature and potential damage to the brain tissue are its major disadvantages [129]. Transplantation into the omentum also provides a highly vascularised and immune‐rich environment for hepatocytes. The omentum exhibits regenerative properties that support cell growth and function. However, this route exhibits some complication risks, such as adhesion and hernia [128, 130]. Direct transplantation of hepatocytes into the liver parenchyma offers the advantage of housing the cells in their natural environment. This route also minimises the risk of immune rejection due to the immune tolerance mechanisms of the liver [131]. However, this procedure is invasive, causing potential damage to the healthy liver tissue [132]. Recent studies have explored the use of lymph nodes as a potential route for hepatocyte transplantation in rodents. Lymphatic system also plays a crucial role in immune surveillance and response, possibly facilitating the integration of administered hepatocytes into the liver tissue. However, this approach has potential disadvantages such as the complexity of targeting specific lymph nodes and the need to ensure the survival and functionality of the administered hepatocytes during their transit through the lymphatic system [133]. One study investigated the use of fat‐associated lymphoid clusters (FALCs) as expandable niches for ectopic liver development and found that hepatocytes transplanted via intraperitoneal injections could engraft into FALCs and form ectopic livers. This study also noted that FALCs offer various advantages over other ectopic liver development sites, such as the ability to expand and contract in response to metabolic demands [134].

### 1.10 Hepatocyte‐Like Cell Implantation in Animal Models

Duan et al. developed a line of HLCs derived from hESCs that displayed an expression of a broad range of liver‐specific genes and proteins. When transplanted into mice, the cells successfully engrafted, survived and secreted human liver‐specific proteins, as detected in mouse serum [135]. The liver engraftment potential of HLCs derived from patient's iPSCs was successfully demonstrated in immunodeficient mouse models and integrated cells maintained their functional characteristics, including albumin secretion and metabolic activity [136]. Moreover, HLCs produced from iPSCs with corrected genetic mutations displayed long‐term survival and recovered expression of liver‐specific functional proteins, such as alpha‐1‐antitrypsin, upon transplantation into animal models [2, 137]. Ang et al. (2018) transplanted the hPSC‐derived liver progenitors in a Fah^−/−^ mouse model of liver failure and demonstrated an improved short‐term survival rate. The study showed key extracellular signalling pathways that led to differentiation and functional maturation of HLCs. Recently, Graffmann et al. provided a comprehensive study on the application of HLCs for modelling various liver diseases and their engraftment capabilities. They highlighted that while HLCs often retain a foetal phenotype in vitro, their maturation and functionality improve significantly after in vivo transplantation [138].

### 1.11 Comparison of Techniques to Stratify the Quality of HLCs

The assessment of HLCs requires a multifaceted approach to ensure their quality and functionality for biomedical applications. Techniques can be categorised into methods for morphological assessment, gene expression and proteomic profiling, functional assays and ultrastructural analyses (Table 7). Morphological assessment methods, such as light and fluorescence microscopy, have moderate specificity as they primarily evaluate cell morphology and attachments. These techniques are moderately sensitive; they can identify general quality but may miss subtle functional differences. High feasibility and low cost are significant advantages of basic microscopy techniques, which are widely accessible and easy to implement in most laboratories [13, 37]. Gene expression profiling techniques, such as qRT‐PCR, and RNA sequencing, offer high specificity by evaluating the presence of liver‐specific markers, thus providing clear insights into the differentiation status of HLCs. These methods are also highly sensitive, as they are capable of detecting low expression levels of genes. However, their feasibility is moderate; while qRT‐PCR can be easily performed, RNA sequencing requires more specialised equipment and expertise. Additionally, they are costly; though qRT‐PCR is relatively affordable, high‐throughput RNA sequencing can be expensive due to the reagents and equipment [25]. Proteomic analysis has high specificity and sensitivity, as it directly assesses the protein expression profiles, which could be correlated with function. However, its feasibility is lower due to the complexity of sample preparation, and data analysis. Furthermore, this approach tends to be expensive due to the need for advanced instrumentation [28, 29, 30, 38]. While functional assays provide moderate to high specificity as they measure direct hepatic functions such as drug metabolism and albumin secretion, these assays are highly sensitive since they can detect functional capabilities that may not be evident through morphological assessments [139]. The feasibility of these assays is moderate; while some assays are straightforward, others may require optimisation and validation for specific applications. Moreover, while basic metabolic assays can be performed inexpensively, more complex assays may involve higher expenses due to reagents and equipment [34, 44, 45, 74, 77]. Three‐dimensional culture systems, such as spheroids and organoids derived from HLCs, offer high specificity by accurately mimicking the liver microenvironment to a certain extent, which enhances HLC functionality assessment. These systems can better reflect in vivo conditions but may still have variability based on culture conditions, so their sensitivity varies from moderate to high. Furthermore, while some 3D culture methods are becoming more standardised, they often require specialised setups that may not be available in all labs. Although basic 3D culture setups can be affordable, complex organoid systems can be expensive due to materials and maintenance requirements [46, 52, 129]. Ultrastructural analysis via TEM provides very high specificity by revealing detailed cellular architecture essential for hepatocyte function and may also identify structural abnormalities. Unfortunately, this technique requires significant investment in equipment and maintenance, along with skilled personnel for sample preparation and imaging [14, 18, 19, 20, 89].

**TABLE 7:** Prioritisation flowchart of techniques for stratifying the quality of HLCs.

	Technical approach	Importance	Specificity	Sensitivity	Feasibility	Cost
Morphological assessment	Light microscopy, fluorescence microscopy	Cell quality, attachment, and differentiation status	Moderate	Moderate	High	Low (basic equipment)
Gene expression profiling	Quantitative RT‐PCR, RNA sequencing	Gene expression profile of liver‐specific and maturation markers	High	High	Moderate	Moderate to high
Proteomic analysis	Mass spectrometry, western blotting, flow cytometry	Protein expression and posttranslational modifications	High	High	Low to moderate	High
Functional assays	Metabolic activity assays, albumin secretion assays, urea production assays	Functional capabilities of HLCs	Moderate to high	High	Moderate	Moderate
3D culture systems	Spheroids and organoids derived from HLCs	Cell–cell interactions and liver architecture	Moderate to high	Moderate to low	Moderate to high	Moderate to high
Ultrastructural analysis	TEM	High‐resolution images of subcellular structures	Very high	Moderate	Low	High

## 2 Conclusion

Hepatocyte‐like cells derived from PSCs are an unlimited source of cells for basic and applied research. Different in vitro and in vivo tests are used to evaluate the functions, maturity and applications of HLCs. These assays must be quality‐controlled and cross‐validated among different laboratories to develop universally accepted criteria for HLC phenotyping. This is important to facilitate the use of HLCs in human drug development, disease modelling and clinical tests [140]. Moreover, future studies should evaluate HLCs in comparison with PHHs, which are the current gold standard.

Utilising a combination of characterisation techniques alongside comparisons with 24‐h cultured primary human hepatocytes (PHHs) is essential for establishing robust criteria for assessing human liver cell (HLC) quality. The criteria established for assessing the quality of HLCs not only enhance our understanding of iPSC‐derived cells but also pave the way for the evaluation of directly reprogrammed HLCs. These directly reprogrammed cells may offer distinct advantages in terms of accessibility and patient‐specific applications [22]. Combined with various cutting‐edge technologies, such as organ‐on‐a‐chip systems, single‐cell analysis and high‐throughput screening, HLCs can improve the in vitro generated tissue definition, performance and phenotypic stability essential for future clinical applications. These advancements are critical for translating HLC research into effective therapeutic strategies for liver diseases. By addressing these aspects, future studies can pave the way for the successful application of HLCs in regenerative medicine and drug discovery.

## Author Contributions

**Zahra Heydari:** writing – original draft (equal), writing – review and editing (equal). **Roberto Gramignoli:** writing – original draft (equal), writing – review and editing (equal). **Abbas Piryaei:** writing – original draft (equal), writing – review and editing (equal). **Ensieh Zahmatkesh:** investigation (equal), methodology (equal). **Paria Pooyan:** investigation (equal), methodology (equal). **Andreas Nussler:** writing – review and editing (equal). **Dagmara Szkolnicka:** writing – review and editing (equal). **Hassan Rashidi:** writing – review and editing (equal). **Mustapha Najimi:** writing – review and editing (equal). **David C. Hay:** conceptualization (equal), supervision (equal), writing – review and editing (equal). **Massoud Vosough:** conceptualization (equal), supervision (equal), writing – review and editing (equal). **Homeyra Seydi:** writing – review and editing (equal).

## Ethics Statement

The authors have nothing to report.

## Consent

The authors have nothing to report.

## Conflicts of Interest

Professor David C. Hay is the founder, director and shareholder of Stimuliver ApS and Stemnovate Limited. Dr. Dagmara Szkolnicka is the founder, director and shareholder of Stimuliver ApS.

## Data Availability

The authors have nothing to report.
